# *Enterococcus faecalis* alters antibiotic susceptibility in *Pseudomonas aeruginosa* mixed-species biofilms

**DOI:** 10.1128/jb.00548-25

**Published:** 2026-04-30

**Authors:** Caleb M. Anderson, Yves Mattenberger, Ana Parga, Heidi Portalier, Casandra Ai Zhu Tan, Maria Esteban Henao, Patrick H. Viollier, Kimberly A. Kline

**Affiliations:** 1 Department of Microbiology and Molecular Medicine, University of Geneva 218785, Geneva, Switzerland; 2 Department of Microbiology and Parasitology, Aquatic One Health Research Center (ARCUS), Faculty of Biology, Universidade de Santiago de Compostela 98707 , Santiago de Compostela, Spain; 3 Singapore Centre for Environmental Life Sciences Engineering, Nanyang Technological University 54761, Singapore, Singapore; Dartmouth College Geisel School of Medicine, Hanover, New Hampshire, USA

**Keywords:** *Pseudomonas aeruginosa*, *Enterococcus faecalis*, polymicrobial, biofilm, antibiotic, antibiotic resistance, iron restriction

## Abstract

Bacterial infections often occur in polymicrobial biofilms where nutrient limitation and interspecies interactions can profoundly shape microbial physiology. *Enterococcus faecalis* can antagonize *Pseudomonas aeruginosa* growth under conditions of iron limitation, such as those found in the mammalian host. Here, we report that this growth antagonism reveals surviving *P. aeruginosa* cells capable of surviving antibiotic challenge, including ampicillin, cefepime, and ciprofloxacin, when grown in iron-restricted biofilms with *E. faecalis*. Transcriptomic profiling of *P. aeruginosa* revealed a distinctive response characterized by broad downregulation of biosynthetic, metabolic, and virulence pathways, alongside selective induction of membrane remodeling proteins, transport systems, and biofilm-associated genes. Induction of *arnT* in *P. aeruginosa*, required for lipid A modification, correlated with enhanced antibiotic survival to ampicillin, cefepime, and ciprofloxacin. Additionally, the diguanylate cyclase SiaD and efflux transporter MfsC in *P. aeruginosa* were implicated in decreased antibiotic susceptibility to the same antibiotics. This transcriptional response was unique to the dual stress of iron deprivation and microbial competition with *E. faecalis*, illustrating how interspecies interactions can simultaneously inhibit and protect *P. aeruginosa*, shedding light on potential persistence mechanisms in iron-limited polymicrobial environments.

**IMPORTANCE:**

This study addresses antibiotic susceptibility in *Pseudomonas aeruginosa*, a major opportunistic ESKAPE pathogen, within polymicrobial biofilms and under host-relevant iron-restricted conditions. Polymicrobial biofilm-associated infections are notoriously difficult to treat due to complex interspecies interactions and increased antibiotic resilience. We demonstrate that *Enterococcus faecalis* not only antagonizes *P. aeruginosa* growth under iron limitation but also induces a unique transcriptional profile, enhancing *P. aeruginosa* survival during antibiotic challenge. This shift involves broad transcriptional reprogramming in *P. aeruginosa*, characterized by global metabolic downregulation and activation of envelope remodeling pathways, including the *arn* operon. These findings reveal how interspecies interactions under iron stress can both suppress and protect bacterial pathogens and underscore the importance of considering community context in treatment strategies for persistent infections.

## INTRODUCTION

Bacteria typically exist within complex communities where interspecies interactions play a critical role in survival, especially under stressful conditions such as nutrient limitation or antimicrobial pressure (1–14). Understanding these interactions is crucial for elucidating mechanisms of microbial persistence, especially in the context of polymicrobial infections (1, 6, 12, 13, 15). Among the organisms frequently implicated in polymicrobial and biofilm-associated infections are *Pseudomonas aeruginosa* (Pa) and *Enterococcus faecalis* (Ef), opportunistic pathogens that exhibit robust resilience to a broad spectrum of antibiotics (2, 16–18). While each organism is independently capable of causing diverse infections, their frequent co-isolation in polymicrobial biofilm-associated infections, including chronic wound infections, periodontitis, and urinary tract infections, supports more careful study of their interspecies interactions in these environments (3, 19–28).

Interactions between *P. aeruginosa* and *E. faecalis* can profoundly alter their individual behavior in an environmental context (29–33). During co-infection in pyelonephritis, *E. faecalis* increases *P. aeruginosa* tolerance to β-lactam antibiotics, suggesting a cooperative effect under specific conditions (29). A different interaction occurs under iron-restricted (IR) conditions in which *E. faecalis* antagonizes *P. aeruginosa* growth, reducing its survival to approximately 0.02% in *in vitro* biofilm assays (33). This antagonism is driven by a combination of competition for iron and environmental acidification caused by *E. faecalis* lactic acid production, illustrating an iron deprivation-induced physiological antagonism between gram-positive and gram-negative cells (33). Iron is an essential nutrient for bacterial growth and is tightly sequestered in the host as a component of nutritional immunity (10, 34, 35). To overcome this limitation, pathogens employ mechanisms such as siderophore production and specialized iron uptake systems adapted to their infection niche (8, 35–37). We previously showed that *E. faecalis* enhances *Escherichia coli* survival under iron deprivation by exporting L-ornithine, which induces enterobactin synthesis in *E. coli* (38). This synergism contrasts with the antagonistic interaction observed *in vitro* between *P. aeruginosa* and *E. faecalis* under iron starvation (33). The molecular responses of *P. aeruginosa* to competition with *E. faecalis* under these conditions remain uncharacterized.

In this study, we elucidated the consequences of interactions between *E. faecalis* and *P. aeruginosa*, focusing on (i) how these interactions influence antibiotic susceptibility under (chemically induced) iron-limiting conditions and (ii) identifying the underlying genetic determinants. Our results reveal that although *E. faecalis* antagonizes *P. aeruginosa* growth, it significantly enhances broad-spectrum antibiotic survival among the remaining *P. aeruginosa* population. Growth with *E. faecalis* under iron restriction reshapes the transcriptome of *P. aeruginosa*, resulting in broad downregulation of essential metabolic, regulatory, stress response, and virulence pathways, while selectively upregulating cell envelope remodeling genes. Among these genes is *arnT*, which mediates the transfer of L-Ara4N to lipid A, a modification known to reduce susceptibility to cationic antimicrobials and thereby confer increased antibiotic tolerance in *P. aeruginosa* (39–42). We show that *arnT* also promotes *P. aeruginosa* survival during exposure to β-lactam and fluoroquinolone antibiotics when grown alongside *E. faecalis* under iron restriction. Likewise, the *P. aeruginosa* c-di-GMP synthase SiaD and the major facilitator transporter MfsC are required for *E. faecalis*-induced antibiotic survival in *P. aeruginosa*, as inactivation of either gene markedly reduced survival following antibiotic challenge across multiple antibiotic classes. Our findings demonstrate that polymicrobial interactions under infection-relevant iron-restricted conditions not only antagonize *P. aeruginosa* growth but also confer an adaptive advantage to the surviving population by altering antibiotic susceptibility.

## RESULTS

### *E. faecalis* and *P. aeruginosa* interactions increase antibiotic tolerance under iron restriction

**Fig 1:**
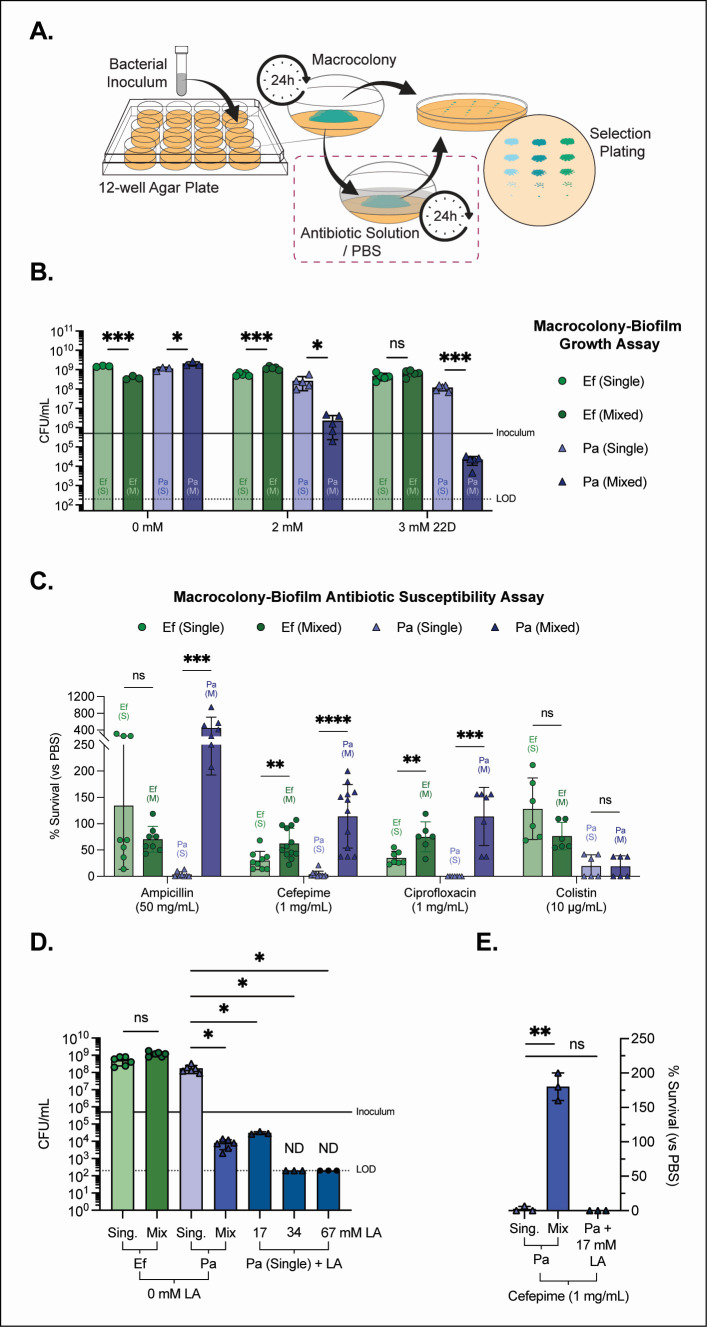
Impact of *E. faecalis* and *P. aeruginosa* interactions on growth and antibiotic tolerance under iron restriction. (**A**) Schematic of the macrocolony growth and antibiotic-challenge assays performed in a 12-well plate format. (**B**) Quantification of bacterial growth as colony-forming units per milliliter (CFU/mL) for *E. faecalis* OG1RF strain (Ef; circles, green) and *P. aeruginosa* PADP6 strain (Pa; triangles, purple) in single-species (S) and mixed-species (M) macrocolonies grown on iron-restricted tryptic soy broth agar (TSB + 10 mM glucose; TSBG) supplemented with the iron chelator 2,2′-dipyridyl (22D; 0, 2, or 3 mM). The solid line denotes the inoculum (5 × 10^5^ CFU/mL), and the dotted line marks the limit of detection (LOD, 200 CFU/mL). (**C**) Percent survival (%) after 24-h (H) antibiotic challenge (ampicillin, 50 mg/mL; cefepime, 1 mg/mL; ciprofloxacin, 1 mg/mL; or colistin, 10 µg/mL) under IR conditions (3 mM 22D TSBG). Each concentration represents tens to thousands of times the planktonic MIC measured in TSBG (see Table S1: 38–75× for ampicillin, ~188–375× for cefepime, ~1,500–6,000× for ciprofloxacin, and ~7.5–15× for colistin). Percent survival was calculated as (CFU antibiotic/CFU PBS [average]) × 100, using the matched PBS-treated control of the same biofilm type (single or mixed), where 100% denotes no change relative to PBS, values <100% indicate antibiotic-mediated killing, and >100% indicate increased CFU recovery after antibiotic compared with PBS. (**D**) Macrocolony biofilm growth assay (CFU/mL) on 3 mM 22D (IR) TSBG agar with and without lactic acid (LA). Ef and Pa in single-IR and mixed-IR macrocolonies are compared with single-IR Pa supplemented with lactic acid (LA; 17, 34, or 67 mM, Pa CFU not detected at 34 and 67 mM [ND] with data points plotted at LOD). LA reproduced the growth antagonism observed in mixed-IR (with Ef) macrocolonies at 17 mM LA but did not confer the increased cefepime survival observed in mixed-IR growth with cefepime challenge (**E**), indicating that acid stress alone is insufficient to recapitulate the Pa antibiotic protective phenotype. Mean ± standard deviation (SD) is shown, and dots indicate biological replicates (*N*) from all performed experiments. Unpaired *t*-tests compare single- vs mixed-species biofilms for each antibiotic within a species. Statistical significance is visualized as: ns, not significant; *, *P* < 0.05; **, *P* < 0.01; ***, *P* < 0.001; and ****, *P* < 0.0001. ND, not detected (≤LOD).

As previously reported, *E. faecalis* strongly antagonizes *P. aeruginosa* growth in iron-restricted macrocolony biofilms in a 2,2′-dipyridyl (22D; iron chelator) dose-dependent manner (Fig. 1A and B) (33). To determine how this interaction affects antibiotic susceptibility, we challenged 24-h iron-starved macrocolonies with high doses of an aminopenicillin (ampicillin), a fourth-generation cephalosporin (cefepime), a fluoroquinolone (ciprofloxacin), or a polymyxin (colistin) antibiotic (Fig. 1A and C) and measured the percent survival (Fig. 1C). To test the antibiotic susceptibility of single- and mixed-species macrocolonies, we used antibiotic concentrations far above the planktonic MICs determined for Pa in tryptic soy broth agar supplemented with glucose (TSBG) (Table S1): ampicillin, 50 mg/mL (~38–75× MIC); cefepime, 1 mg/mL (~188–375× MIC); ciprofloxacin, 1 mg/mL (~1,500–6,000× MIC); and colistin, 10 µg/mL (~7.5–15× MIC), using the MIC ranges obtained for Pa strains PAO1 and PADP6 (a 22D tolerant strain of PAO1) (33). These supralethal doses were selected to drive near-complete killing in Pa single-species macrocolonies, thereby sensitizing the assay to detect any robust protective effects of mixed-species growth with Ef.

In iron-restricted (IR) mixed biofilms with Pa, Ef survival increased compared to single-species biofilms when challenged with cefepime (62% mixed-IR survival vs 30% single-IR) or ciprofloxacin (75% mixed-IR vs 35% single-IR). Ampicillin and colistin produced non-significant reductions in Ef survival in the mixed biofilm (134% single-IR vs 70% mixed-IR and 128% single-IR vs 76% mixed-IR, respectively). Upon ampicillin challenge, Pa single-species biofilms (IR) were reduced to ~4% survival compared to their PBS control, near the limit of detection (LOD), whereas mixed IR biofilms yielded ~452% survival, reflecting an ~4.5-fold increase in CFU compared to the PBS control. Importantly, IR-Ef CFUs were not markedly reduced by ampicillin in the mixed IR biofilms, indicating that enhanced Pa survival under this condition is not generally explained by antibiotic-mediated elimination of Ef. After cefepime exposure, Pa single-species biofilms (single-IR) were reduced to ~4% survival, whereas mixed-IR biofilms yielded ~128% survival relative to the PBS control. Similarly, after challenge with ciprofloxacin, IR single-species biofilms were reduced to undetectable levels, while mixed biofilms yielded ~114% survival relative to the PBS control, reflecting an ~1.1-fold increase. This enhanced survival phenotype was not observed with colistin treatment (mixed-IR vs single-IR), with no statistically significant difference in Pa colistin susceptibility observed between single- and mixed-species IR conditions. Absolute CFU enumerations used for percent survival calculations can be seen in Fig. S1.

Because Ef-dependent antagonism under iron restriction involves lactic acid production, we asked whether acid stress alone could drive the antibiotic susceptibility phenotype (33). Supplementing Pa single-species biofilms with lactic acid reproduced the growth-inhibitory effect seen in mixed biofilms as previously reported (Fig. 1D) (33) but failed to confer increased survival to cefepime (Fig. 1E). Thus, while lactic acid contributes to growth antagonism, it is insufficient to account for the observed increase in Pa survival under these conditions.

Together, these data show that IR growth with Ef generally antagonizes Pa growth (Fig. 1B) yet confers a protective advantage to the surviving Pa population when challenged with β-lactams or ciprofloxacin at doses far exceeding planktonic MICs. Colistin is a notable exception, consistent with outer-membrane-targeted killing and the potential outer-membrane stress signature we describe below. Finally, this unique state cannot be recreated by lactic acid alone (Fig. 1D and E), suggesting that factors other than acid stress, likely additional Ef-derived metabolites or signals, are required to elicit antibiotic susceptibility changes in Pa under mixed-species, iron-limited biofilm growth conditions.

### Mixed-species ampicillin protection of *P. aeruginosa* is localized to the microcolony biofilm

**Fig 2:**
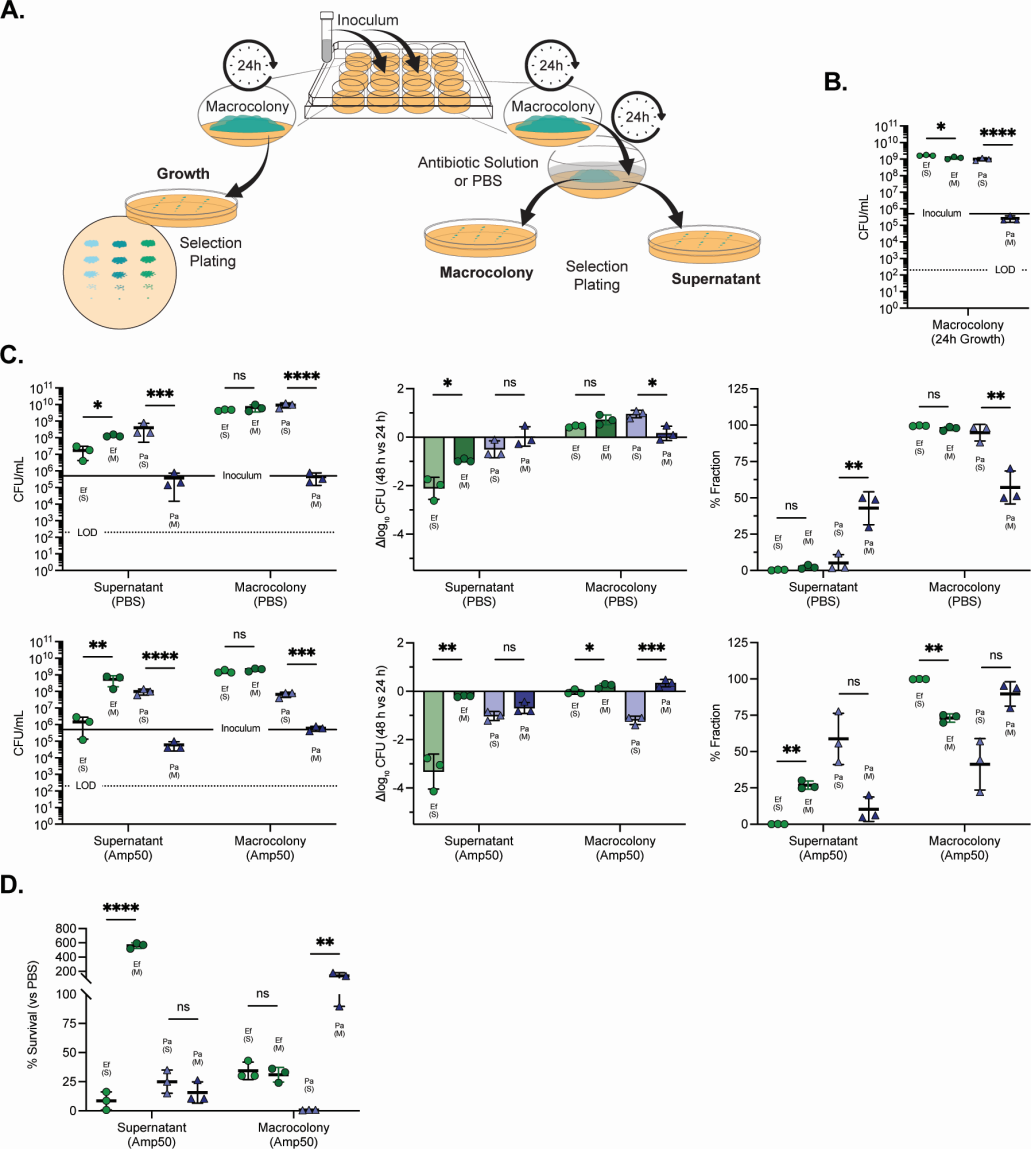
Community-dependent partitioning and ampicillin response of supernatant and macrocolony-associated cells. (**A**) Experimental schematic where *E. faecalis* and *P. aeruginosa* were inoculated onto iron-restricted 22D TSBG agar (tryptic soy broth agar supplemented with 3 mM 2,2′-dipyridyl and 10 mM glucose) and grown as macrocolonies for 24 h. Macrocolonies were then overlaid with PBS or ampicillin (Amp50; 50 mg/mL in PBS) for an additional 24 h. The supernatant overlay fraction was collected without disrupting the macrocolony, and the remaining macrocolony biomass was recovered by total homogenization. Species-specific CFUs were quantified by selection plating. Paired replicates were taken without PBS or antibiotic overlay to determine the growth dynamics at 24 h. (**B**) Baseline macrocolony CFU/mL after 24 h growth, prior to overlay. (**C**) Recovery after 24 h overlay with PBS (top row) or Amp50 (bottom row). Left, CFU/mL recovered from supernatant and macrocolony fractions at 48 h. Middle, change relative to the 24 h baseline expressed as Δlog_10_CFU (48 vs 24 h) {calculated as log_10_(CFU[48 h, fraction]) − log_10_(CFU[24 h, macrocolony])}. Right, compartment distribution expressed as percent fraction of total recovered CFU at 48 h (supernatant/[supernatant + macrocolony] × 100 and macrocolony/[supernatant + macrocolony] × 100). (**D**) Percent survival after Amp50 exposure compared to PBS, calculated separately for supernatant and macrocolony fractions as (Amp50 CFU/mean PBS CFU × 100). Values >100% indicate higher CFU recovery after Amp50 than PBS. Data are shown for Ef (green circles) and Pa (purple triangles) grown as single-species (S) or mixed-species (M; Ef + Pa, 1:1 inoculum) macrocolonies. In panels B and C, the solid horizontal line indicates the starting inoculum (5 × 10^5^ CFU/mL), while the dotted line indicates the limit of detection (LOD; 200 CFU/mL). Bars denote mean ± SD with individual biological replicates overlaid. Statistical significance reflects comparisons of single vs mixed within each organism for the indicated fraction or condition using unpaired two-tailed *t* tests (with log_10_-transformed CFU/mL where shown); ns, not significant; *, *P* < 0.05; **, *P* < 0.01; ***, *P* < 0.001; and ****, *P* < 0.0001.

Because macrocolony antibiotic susceptibility testing is performed by applying antibiotics as a liquid overlay, recovery after treatment reflects bacteria that remain associated with the macrocolony as well as bacteria present in the overlay (i.e., released from the biofilm) at the end of the exposure period. To determine whether differential partitioning into the overlay supernatant could contribute to the mixed-species antibiotic survival phenotypes observed under iron restriction, we fractionated the overlay to differentiate between those cells that migrated or dispersed to the supernatant after 24 h and the macrocolony biomass, enumerating survival separately following 24 h exposure to PBS or ampicillin (50 mg/mL in PBS) (Fig. 2A). After confirming the antagonism of Pa under IR, mixed-species growth with Pa at 24 h (Fig. 2B), the fractionated CFUs were enumerated after 24-h exposure to PBS (Fig. 2C, right, top) or ampicillin (Fig. 2C, right, bottom). Both Ef and Pa were recoverable from the supernatant fraction after PBS overlay, confirming that dispersal occurs during the exposure period (Fig. 2C, right). Under PBS exposure, Ef populations were predominantly macrocolony-associated in both single- and mixed-species macrocolonies, with a small fraction of recovered Ef detected in the supernatant overlay (Fig. 2C, right, top). By contrast, Pa showed clear condition-dependent partitioning. In Pa single-species macrocolonies under iron restriction, Pa remained mostly macrocolony associated (Fig. 2C, left and right, top). However, in mixed macrocolonies, despite Ef antagonism that reduced total Pa abundance, the remaining Pa subpopulation was distributed more evenly between the supernatant and macrocolony fractions, resulting in a higher supernatant fraction for Pa in the mixed-species condition (Fig. 2C, right, top). These results confirm that supernatant-associated, or dispersed, cells are present at the end of the overlay period and that community context can shift the relative partitioning of Pa between compartments, even in the absence of antibiotic.

After ampicillin exposure, Pa exhibited compartment-specific outcomes that clarified the origin of the mixed-species protection phenotype. In Pa IR, single-species biofilms, ampicillin strongly reduced macrocolony-associated cells and increased recovery from the supernatant overlay (Fig. 2C). When changes were visualized relative to the 24-h baseline (Δlog_10_ CFU; 48 vs 24 h), Pa in the single-species condition showed reduced recovery in the macrocolony fraction after ampicillin treatment (Fig. 2C, middle, bottom). By contrast, in mixed-species macrocolonies, Pa recovery after ampicillin exposure remained largely macrocolony-associated, with fewer supernatant overlay or dispersed recovery and minimal net loss relative to the 24-h baseline in the macrocolony fraction (Fig. 2C, middle, bottom). To test whether the mixed-species protection phenotype specifically reflected survival of dispersed cells compared to macrocolony-associated cells, the percent (%) survival relative to matched PBS controls, separated by fraction, was calculated (Fig. 2D). Dispersed Pa survival after ampicillin exposure did not differ between single- and mixed-species macrocolonies, while macrocolony-associated Pa survival increased in the mixed-species condition, reflective of the protective phenotype described above. These results demonstrate that Ef-dependent ampicillin protection under iron restriction is limited to the macrocolony-associated Pa population and is not explained by preferential dispersal into the supernatant overlay.

### Combined iron limitation and interspecies competition induce a unique transcriptional response in *P. aeruginosa*

**Fig 3:**
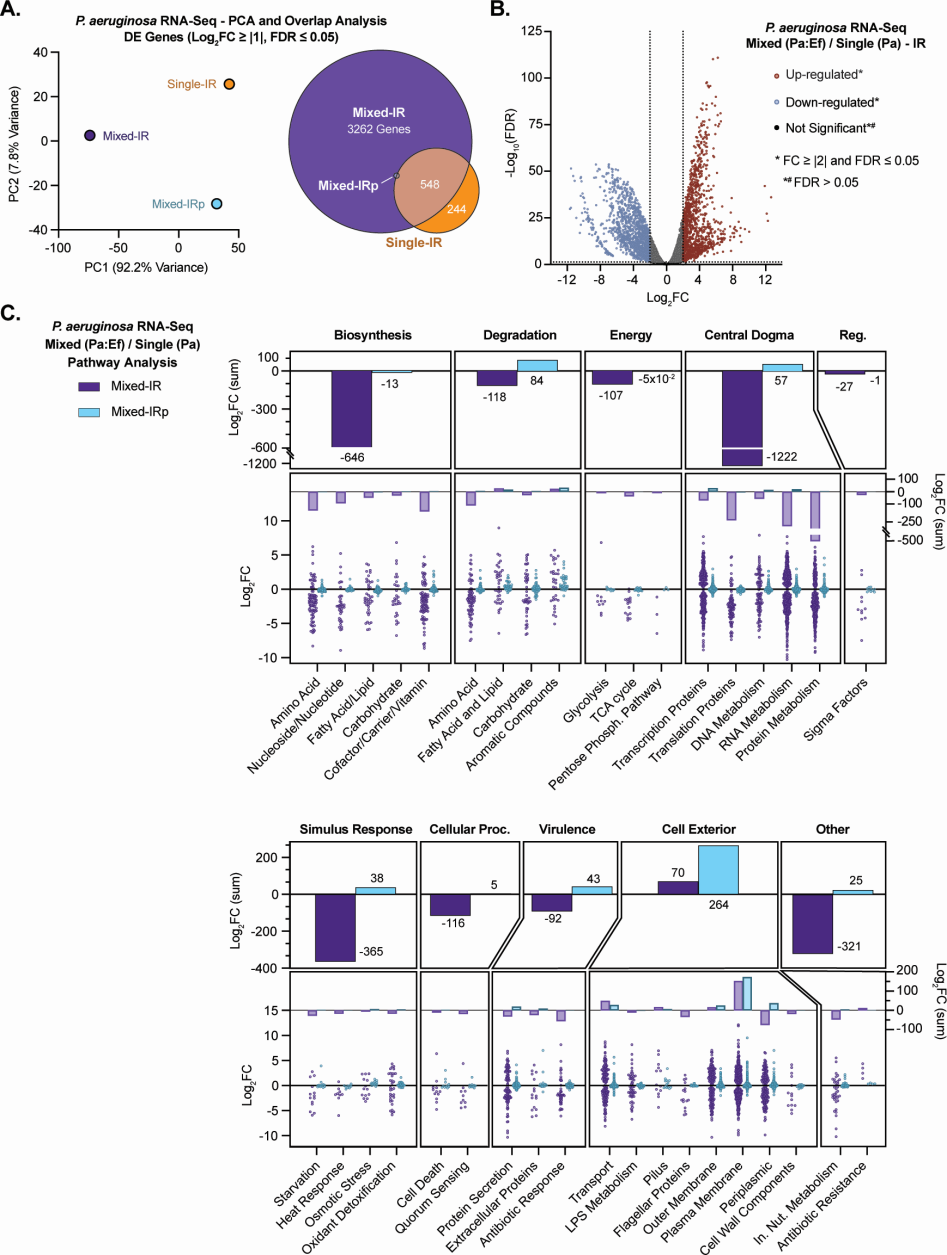
Global transcriptional reprogramming of *P. aeruginosa* under iron-restricted co-culture with *E. faecalis*. (**A**) PCA plot (left) of significantly differentially expressed *P. aeruginosa* genes (FDR ≤ 0.05) in at least one of the three pairwise comparisons from the RNA-seq analysis of macrocolony biofilms under iron restriction (single-IR vs single-IRp, mixed-IR vs single-IR, and mixed-IRp vs single-IRp), with each point representing the log_2_FC profile for one comparison. A Venn diagram (right) shows the overlap analysis of the subset of these genes with |log_2_FC| ≥ 1 (FDR ≤ 0.05), with the number of unique and overlapping genes in each data set indicated. The mixed-IRp condition contained two genes in common with mixed-IR and two genes in common with single-IR/mixed-IR. See Table S3 for gene lists. (**B**) Volcano plot visualizing differentially expressed genes in Pa mixed-IR (with Ef) vs single-IR. (**C**) Pathway analysis (BioCyc) of statistically significant (FDR ≤ 0.05) differentially expressed genes in Pa mixed-IR (with Ef) vs single-IR and mixed-IRp (with Ef) vs single-IRp conditions. Graphs show general pathways as the sum of the log_2_FC values for the genes in that pathway (top bar graph), followed by the log_2_FC sum values for the genes in the subpathways (middle bar graph), and finally the individual log_2_FC values for the genes in each corresponding subpathway (bottom dot plot). This multi-tiered design displays pathway-level log_2_FC sums interpreted in context, as individual gene-level changes, including potential outliers, are visualized in the accompanying subpathway and gene-level panels. See Table S4 for pathway gene lists.

RNA sequencing of *P. aeruginosa* and *E. faecalis* in single- and mixed-species macrocolony biofilms under iron-restricted and iron-replete (IRp) conditions revealed extensive, context-dependent transcriptional remodeling. Ef showed a minimal transcriptional response to mixed-IR growth with Pa, with only 23 genes significantly upregulated and 35 downregulated (mixed-IR vs single-IR). These differentially expressed genes included modest upregulation of myo-inositol and carbohydrate utilization genes together with selected membrane and transporter loci, while high-affinity phosphate transport and thiamine biosynthesis genes were downregulated (Table S2). Notably, many of these genes were differentially expressed in the same direction, consistently upregulated or downregulated. When comparing mixed- vs single-species Ef biofilms under IRp conditions, larger amplitudes of expression changes were observed in mixed-IRp vs single-IRp biofilms, indicating that iron restriction reduces the magnitude of the Ef mixed-species response. Evaluating iron restriction alone in Ef (single-IR vs single-IRp), we observed a comparatively greater transcriptional response (471 genes significantly upregulated and 469 downregulated), enriched for metal and iron acquisition and transport genes such as *adcA, feoB*, and multiple ABC transporters, as well as lactate metabolism genes *larABCD* and inositol catabolism genes (Table S2). Under IR conditions, mixed-species biofilm growth with Ef triggered the most extensive changes in Pa, whereby approximately 73% of Pa genes were differentially expressed (mixed-IR vs single-IR) (Table S2) (FDR ≤ 0.05 and log_2_FC > |1|). Iron restriction alone (single-IR vs single-IRp) induced differential expression for 15% of genes in Pa, and fewer than 7% of Pa genes were differentially expressed in mixed-species biofilms compared to single-species growth when iron was not restricted (mixed-IRp vs single-IRp). Principal component analysis (PCA) of Pa single-IR (vs single-IRp), mixed-IR (vs single-IR), and mixed-IRp (vs single-IRp) revealed the distinctiveness of the mixed-IR samples, which separated along PC1 (92.2% variance), indicating the influence of combined iron scarcity and Ef competition on Pa transcription (Fig. 3A).

In mixed-IR vs single-IR conditions, Pa exhibited a global adaptive response characterized by broad metabolic downregulation and selective upregulation of nutrient uptake and transport pathways (such as *mfsC*, log_2_FC +9.04), surface remodeling pathways such as envelope modifications, and community-associated pathways involved in biofilm formation, adhesion, and quorum-sensing regulation, represented under the Virulence and Stimulus response categories (e.g., *pelA*, *algX*, *arr*, and *cupB1*) and discussed more below (Fig. 3C; Table S4). The largest affected groups included cytoplasmic membrane-associated genes, reflecting shifts in membrane composition and transport functionality, with a hallmark of the transcriptomic response in the Pa mixed-IR biofilms being extensive suppression of energetically demanding processes (Fig. 3C). Biosynthesis pathways for amino acids, vitamins, fatty acids, and isoprenoids were notably downregulated, as were nucleotide biosynthesis pathways (mean log_2_FC/pathway: purine, −2.53; pyrimidine, −3.47). Central dogma machinery, particularly ribosomal proteins (*rpsU*, −7.36; *rpsR*, −6.90; and *rpmH*, −6.85), tRNA processing factors, and sigma factors (*rpoS*, −4.2; *sigX*, −4.4; and *algU*, −1.2), were downregulated, resulting in proposed reduced protein synthesis capacity and growth. Additionally, energy-generating pathways, including glycolysis, Entner-Doudoroff, oxidative pentose phosphate pathways, and the TCA cycle, were downregulated (Table S4). Iron-dependent respiratory complexes similarly decreased, whereas fermentative enzymes and non-iron oxidoreductases were modestly induced, suggesting alternative metabolic routes.

Evaluating the general response to iron restriction, single-IR Pa biofilms (vs single-IRp) predominantly activated canonical iron acquisition systems, such as siderophore pyochelin and pyoverdine synthesis genes (e.g., *pchA* to *pchG* gene clusters and *pvdD*, *pvdE*, *pvdH*, *pvdI*, and *pvdJ*), as well as endogenous siderophores, heme and ferrous iron OM and/or IM transporter genes (e.g., *fptA* and *fptX; fpvA; hasR* and *hasAp*; *phuR;* and *feoB*) (Table S2). Surprisingly, this canonical iron starvation response in Pa was largely suppressed in the presence of Ef (mixed-IR vs single-IR)*,* indicating that Ef significantly interfered or competed with Pa iron-regulated processes. Several genes coding for OM and IM xenosiderophore transporters showed compensatory induction (e.g., *optJ*, *+*6.65; *foxB*, *+*5.84; *fepG*, *+*3.18; *fvbA*, *+*2.79; *fepB*, *+*2.44; *foxA*, *+*2.41; *pfuA*, *+*2.30; *fiuB*, *+*2.25; *PA1909*, *+*2.14; *fecA*, *+*1.72; *pirA*, *+*1.64; and *piuA*, *+*1.56).

Despite widespread metabolic downregulation in the mixed-IR condition, Pa pathways related to nutrient uptake and community sensing were selectively activated compared to single-IR growth (Fig. 3C; Tables S2 and S4). Genes for inorganic ion and carbohydrate transporters, particularly ABC and P-type ATPase transporters (*PA1429,* +7.81; *PA3383,* +6.61; and *nasA*, +5.40), were strongly induced (mixed-IR vs single-IR), reflecting potential nutrient scavenging adaptations. Efflux systems for heavy metals and toxins (*czcA*, +5.02; *czcB*, +3.78) were also upregulated, possibly to enhance environmental resilience. Additionally, the upregulation of Pa biofilm formation-associated genes was a response uniquely observed under mixed-IR growth (compared to single-IR), reflected in the marked induction of matrix production and adhesion genes (*pelA*, +4.96; *algX*, +4.95; *arr*, +4.83; and *cupB1*, +4.23). Upregulation of Pa biosynthesis and cyclic di-GMP signaling genes (*siaD*, +7.03; *pelD*, +4.01; *alg44,* +4.16; and *bfiS*, +2.65) in the mixed-IR vs single-IR condition reflects a potential shift toward more sessile, community-oriented lifestyle adaptations. Pa biofilm regulation in the single-IR condition was more variable and limited, further emphasizing the specificity of coordinated biofilm induction in the presence of Ef.

Under mixed-IR growth (vs single-IR), Pa genes associated with lipid A modification via the *arn* operon (*arnBCADT,* all upregulated *+*1.42 *to +*8.17) and the *eptA* gene (+6.23), both known to mediate resistance to antimicrobial peptides, were notably upregulated. The Pa global regulator *ampR* (+2.51), associated with resistance and stress responses, was similarly upregulated (mixed-IR vs single-IR), suggesting targeted adaptive resistance mechanisms in Pa in response to Ef competition. Traditional antibiotic-response genes, found in Fig. 3C and Table S4, however, were mostly downregulated under the same conditions, emphasizing the specificity of these adaptations (Fig. 3C). Virulence-associated pathways also showed differential modulation, with general repression of flagella, Type II and III secretion systems, yet selective induction of adhesion factors (Table S2). Quorum-sensing pathways experienced marked repression of signal synthesis genes (*pqsH*, −4.45), with selective upregulation of specific regulatory genes (*vqsM*, +4.38), indicating a shift toward energy-conserving, responsive behaviors.

Collectively, mixed iron-restricted growth with Ef, not iron restriction alone, drives a unique and specialized Pa transcriptional response that conserves resources, redirects nutrient acquisition, reinforces the cell envelope, and promotes biofilm formation in this polymicrobial, iron-limited environment.

### Mixed-species-dependent decrease in *P. aeruginosa* antibiotic susceptibility under iron restriction requires ArnT, PmrA, SiaD, and MfsC

**Fig 4:**
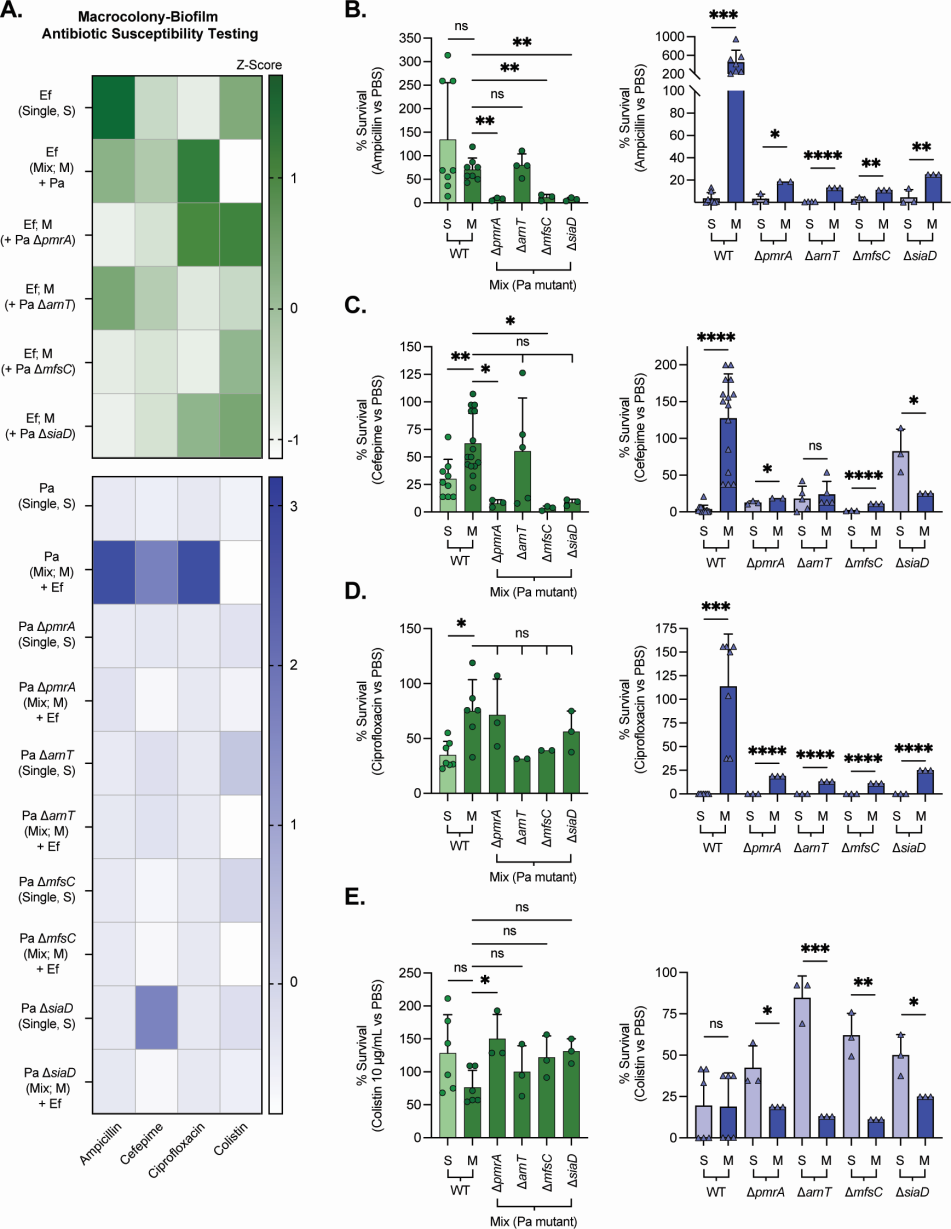
Mixed-species-dependent decrease in *P. aeruginosa* antibiotic susceptibility under iron restriction requires ArnT, PmrA, SiaD, and MfsC. (**A**) Macrocolony-biofilm antibiotic susceptibility heatmaps (iron-restricted medium). Top, *E. faecalis* (Ef; green), shows average percent survival relative to PBS controls for Ef grown alone or mixed with *P. aeruginosa* (Pa) WT or the indicated Pa mutants (Δ*pmrA*, Δ*arnT*, Δ*mfsC*, and Δ*siaD*). Bottom, Pa (purple), shows average percent survival for Pa grown alone or with Ef for WT and the indicated Pa mutants. Antibiotic challenges of 24-h macrocolony biofilms for 24 h with ampicillin (50 mg/mL), cefepime (1 mg/mL), ciprofloxacin (1 mg/mL), or colistin (10 µg/mL). Color scales represent *z*-scores of the mean survival values across strains and conditions by antibiotic. Mixed-species biofilm growth broadly shifts Pa toward higher survival after β-lactam and ciprofloxacin exposure, whereas colistin sensitivity increases compared to the Pa mutant single-species conditions. (**B–E**) Bar graphs show percent survival (antibiotic/PBS) for Ef (left panels, green circles) and Pa (right panels, blue triangles) under the same conditions for (**B**) ampicillin, (**C**) cefepime, (**D**) ciprofloxacin, and (**E**) colistin. Relative to monospecies growth, WT Pa in mixed macrocolonies with Ef exhibited a marked increase in survival to ampicillin, cefepime, and ciprofloxacin, with mixed-species protection lost in Pa mutants for Δ*pmrA* and Δ*arnT* (L-Ara4N lipid A pathway) and in Δ*siaD* (c-di-GMP synthase) and Δ*mfsC* (major facilitator transporter). Colistin showed no mixed-species protection for Pa across all genotypes. Ef survival showed greater variation depending on antibiotic and Pa genotype. Bars show means ± SD with individual biological replicates overlaid (*n* values, individual biological replicates, shown as overlaid data points), and survival values were normalized to paired PBS-treated macrocolonies, with statistical significance determined via unpaired two-tailed *t* tests or one-way ANOVA for Ef + Pa mutants (meaning Ef + Pa WT mix vs each individual Ef + Pa mutant mix); ns, not significant; *, *P* < 0.05; **, *P* < 0.01; ***, *P* < 0.001; and ****, *P* < 0.0001. WT single- and mixed-species values shown are the same data set presented in Fig. 1C, replotted here for direct comparison with the mutant strains.

To define the mechanisms underlying the decreased antibiotic susceptibility of *P. aeruginosa* observed in *E. faecalis* mixed-species biofilms grown under iron-restricted conditions (Fig. 1C), we assessed antimicrobial susceptibility in Pa isogenic mutants lacking genes identified by RNA sequencing as potential contributors to this phenotype in the mixed-IR vs single-IR data set (Table S2). All susceptibility tests were performed under IR conditions using the same antibiotic concentrations as in Fig. 1C: ampicillin, 50 mg/mL (~38–75× MIC in TSB); cefepime, 1 mg/mL (~188–375×); ciprofloxacin, 1 mg/mL (~1,500–6,000×); and colistin 10 µg/mL (~7.5–15×). Percent survival for each condition was calculated relative to the matched PBS control of the same macrocolony biofilm type as described above. Heatmaps of replicate macrocolony assays (*z*-scores) (Fig. 4A) for antibiotics across strains show that, consistent with Fig. 1C, mixed-IR Pa survival increased after cefepime, ampicillin, and ciprofloxacin challenge compared to single-IR, but with no protection against colistin (Fig. 1C, 4B, and C). Transcriptomic analyses identified induction of lipid A remodeling, efflux transporters, and biofilm-associated genes in Pa, uniquely in response to combined dual Ef competition and iron limitation. Accordingly, we investigated their contribution to the altered antimicrobial susceptibility phenotype using Pa isogenic mutants lacking ArnT, responsible for the addition of L-Ara4N to lipid A (39), the regulator of the *arn* operon PmrA (43), the positive regulator of biofilm formation SiaD (44, 45), and the efflux pump component MfsC (46). Loss of *arnT*, *pmrA*, *siaD*, or *mfsC* abolished the increased survival observed for ampicillin, cefepime, and ciprofloxacin. For colistin, the mutants exhibited increased survival in single-IR growth, possibly pointing toward other lipid A modifications that protect cells efficiently against colistin (Fig. 4B through E). For Ef, antibiotic survival was largely unchanged by Pa genotype, although statistically significant decreases in Ef survival were observed in mixed-species biofilms of Ef with Pa Δ*arnT* (Δ*pmrA*), Δ*mfsC*, and Δ*siaD* during ampicillin challenge, with Pa Δ*pmrA* and Δ*mfsC* for cefepime, and Pa Δ*pmrA* after colistin treatment, all compared to Ef-Pa wild-type (WT) mix (Fig. 4B through E). Collectively, these data demonstrate that the Ef-dependent decrease in Pa antibiotic susceptibility under iron restriction is an active, genetically encoded, and multifactorial response, requiring at minimum ArnT-mediated lipid A modification together with SiaD-dependent c-di-GMP signaling and the transporter MfsC, highlighting that mixed species Ef and Pa colocalization under iron limitation engages specific regulatory circuits that subsequently enhance antibiotic survival.

## DISCUSSION

Host-imposed iron restriction is a defining feature of many infection niches, but its impact on bacterial physiology in polymicrobial communities remains largely unexplored (13). Mixed microbial communities, common in wounds, lungs, and urinary tracts, face the dual constraints of nutritional immunity and interspecies competition, and these combined stresses can fundamentally alter microbial dynamics, altering growth, virulence, and antibiotic susceptibility (29, 33, 47, 48). Cooperative effects between *Enterococcus faecalis* and *Pseudomonas aeruginosa* have previously been noted *in vivo*, where Ef coinfection increased Pa tolerance to β-lactam therapy in a murine pyelonephritis model (29), and Ef is known to facilitate both synergistic and antagonistic interactions with cohabitating bacterial species such as *E. coli* and *Staphylococcus aureus* depending on environmental context (15, 38, 49–56). In this study, we define how Pa adapts to biofilm growth with Ef under iron restriction and how interactions between these two pathogens, specifically under iron restriction, influence antibiotic susceptibility. Consistent with prior work, Ef strongly antagonized Pa growth in mixed iron-restricted macrocolony biofilms, reducing viable Pa by >99% relative to Pa iron-restricted single-species biofilms (33), yet the surviving antagonized Pa subpopulation displayed significantly increased survival to several antibiotics (ampicillin, cefepime, and ciprofloxacin), at doses far exceeding planktonic minimum inhibitory concentrations, while colistin remained broadly bactericidal. Our findings support the concept that antagonism and stress can drive community-dependent protective states that are not predicted by single-species or single-stressor measurements.

Robust iron restriction-associated transcriptional changes in metal transport and metabolism were observed in Ef when comparing its single-species growth under iron restriction to that without iron limitation, consistent with iron restriction as a strong environmental driver of adaptive responses. However, Ef transcriptomic responses were comparatively modest in mixed- vs single-species biofilms under iron restriction, suggesting that Ef may be relatively unaffected by Pa in this environment, or that its primary impact on the community in this context is mediated through metabolic outputs rather than dramatic transcriptional remodeling. These data were collected in the absence of antibiotics, and we therefore cannot exclude that antibiotic treatment induces additional changes, transcriptional, post-transcriptional, or metabolic, in Ef not reflected in recovered growth dynamics, which could secondarily influence its antagonistic or protective effects on Pa.

By contrast, Pa displayed extensive transcriptional remodeling specifically in the mixed iron-restricted condition, consistent with Ef-imposed antagonism and iron starvation jointly reshaping Pa physiology. Pa in the Ef-mixed-iron restricted condition adopted an expression profile that diverged sharply from both its canonical iron-starvation response in single-species biofilms and from mixed biofilms grown under non-iron-restricted conditions, reflective of a broad stress-adapted state characterized by downregulation of growth-related metabolism and canonical iron acquisition pathways, consistent with an energy-conserving, slow-growth state (57, 58), alongside upregulation of multiple defensive pathways. This pattern suggests an active survival strategy rather than passive starvation, where high-cost, iron-intensive processes were downregulated, while less iron-dependent metabolic routes were selectively induced (59, 60). Similar metabolic downshifting has been linked to antibiotic tolerance through reduced activity of antibiotic targets and limiting reactive oxygen production, which could be helping antagonized surviving Pa endure nutrient deprivation while also inducing antibiotic recalcitrance (57, 58, 61–63). We observed upregulation of transport and stress-response systems in Pa under mixed-iron-restricted growth, including ion and small molecule transporters, heavy metal efflux components (*czcABC*), and multidrug efflux regulators, mechanisms broadly associated with biofilm-associated antibiotic tolerance (64). This broader stress-adapted state also included signatures of cell envelope remodeling, suggesting Pa is not only slowing growth but also actively reinforcing barrier and defense functions in response to this polymicrobial environment (60, 65).

One of the strongest Pa transcriptional responses in mixed-iron restricted macrocolonies was the induction of the *arn-*encoded lipid A modification pathway and the phosphoethanolamine transferase gene *eptA*, consistent with envelope remodeling that can plausibly alter susceptibility to antibiotics and host antimicrobials. The *arn* operon mediates the addition of 4-amino-4-deoxy-L-arabinose (L-Ara4N) to lipid A (42, 65), a modification that reduces outer-membrane negative charge and is associated with resistance to cationic antimicrobial peptides (CAMPs), including polymyxins (42). In Pa, *arn* expression is controlled by the PhoPQ and PmrAB two-component systems under divalent cation limitation (60). Genetic tests indicated that envelope remodeling was necessary for the altered antibiotic susceptibility in Pa observed in mixed-iron restricted biofilms, whereby deletion of *arnT* (L-Ara4N transferase) or *pmrA* eliminated the mixed-species survival phenotype of Pa to β-lactams and ciprofloxacin, arguing that mixed-iron restricted growth drives a functionally relevant remodeling that impacts antibiotic susceptibility, extending beyond CAMPs. This broader consequence is consistent with LPS remodeling altering permeability, drug access, or envelope stability even for antibiotics that do not directly target LPS and is supported by evidence in other gram-negative species, where *arn*-dependent remodeling contributes to tolerance against β-lactams such as carbapenems (66). Importantly, *arn* induction was not a default consequence of iron starvation in Pa single-species biofilms, reinforcing that the phenotype is contextually driven by polymicrobial growth under iron restriction.

In addition to envelope remodeling, mixed-iron restricted growth elevated the expression of Pa biofilm-matrix genes and regulatory pathways, including *pel, alg, cup,* and the diguanylate cyclase *siaD*, consistent with a shift toward a c-di-GMP-driven biofilm regulatory state. Elevated intracellular c-di-GMP has been shown to promote biofilm formation and antibiotic tolerance by decreasing growth rate, enhancing matrix production, and coordinating stress defenses (67–74). Functional testing again supported a role in antibiotic protection in Pa, where *siaD* (71) deletion abrogated the survival advantage conferred by Ef under iron restriction, reinforcing the importance of the biofilm regulatory state and matrix-associated defenses in this model. Consistent with a multifactorial tolerance program, loss of the major facilitator transporter MfsC (64) also eliminated the mixed-species survival phenotype, supporting a contribution of efflux to decreased antibiotic susceptibility. Together, these results argue that Ef under iron restriction does not simply protect Pa from antibiotics, but rather induces a multicomponent Pa defense response that integrates envelope remodeling (*arn* and *eptA*) biofilm adaptation and regulation (*siaD*), and efflux (*mfsC*). Beyond transcriptional regulation, the matrix itself can provide protection by limiting antibiotic diffusion and through the binding of antibiotics (68, 75). While we did not directly quantify matrix composition here, the combined genetic requirement for *siaD* and the coordinated induction of multiple matrix loci support a model in which matrix-mediated barriers act in concert with envelope remodeling and efflux rather than as a single dominant mechanism (64, 68).

The spatial localization of this survival phenotype further supports a biofilm-embedded mechanism. Macrocolony susceptibility testing used a liquid suspension overlay, where dispersal into the overlay could confound apparent killing or survival if dispersed cells differed in antibiotic susceptibility. Fractionation of the supernatant overlay confirmed that dispersal does occur during the assay, but the mixed-species survival advantage to ampicillin was limited to the macrocolony-associated cells, where dispersed Pa survival did not significantly differ between single- and mixed-species conditions. Arguing against preferential dispersal as the primary explanation for the protection in mixed-species conditions, we report a model in which the protective state is enriched within the biofilm-associated Pa subpopulation, consistent with the unique response state described above.

The transcriptional suppression of canonical Pa iron acquisition pathways during mixed-iron restricted growth provides additional insight into how Ef reshapes the microenvironment to favor survival over iron scavenging. In Pa single-species biofilms, iron starvation typically induces siderophore production and iron-uptake systems (37, 76–79), yet many of these were downregulated during mixed-iron-restricted growth despite severe iron limitation. Ef is known to undergo fermentative metabolism under iron restriction, exporting L-lactate that acidifies the milieu and chelates iron, and we previously showed an Ef Δ*ldh1* mutant, defective in lactic acid production, loses the ability to antagonize Pa growth (33). These environment changes could render siderophore-mediated iron scavenging less effective for Pa, thereby selecting for energy conservation and stress tolerance rather than continued investment in iron acquisition (33). Lactic acid alone was insufficient to reproduce the increased antibiotic survival phenotype in Pa, despite recapitulating the growth inhibition observed under iron restriction (33). This indicates that additional cues generated during cohabitation with Ef, potentially including contact-dependent signaling, peptide or small molecule effectors, or redox or ionic changes, are required to drive the full protective state (17, 80–87). Identifying which signals are necessary and sufficient will be important for defining how Pa prioritizes survival pathways over iron scavenging during polymicrobial stress.

The mixed-iron-restricted transcriptome of Pa also featured strong induction of *ampR*, a LysR-type regulator with broad regulon effects, including β-lactamase (*ampC*), efflux, quorum sensing, and virulence factors (88). Although AmpR can repress biofilm formation under some conditions, our data showed simultaneous upregulation of biofilm gene sets, implying that c-di-GMP signaling and iron-stress networks may override or redirect AmpR outputs in this dual-stress setting (88). Because AmpR integrates cell wall and envelope stress signals, its induction may help coordinate the *arn*/efflux arm of the response, while other regulators such as c-di-GMP circuits or stringent response drive matrix-associated tolerance and growth arrest (61, 88). Moreover, reduced growth or cell cycle arrest of Pa in mixed-iron-restricted biofilm growth might, directly or indirectly, alter the pool of peptidoglycan-derived ligands that modulate AmpR activity, since specific peptidoglycan degradation products imported into the cytoplasm act as activators of AmpR, while a peptidoglycan biosynthetic precursor promotes repression (89). Testing *ampR* mutants in this model will be useful to clarify whether AmpR is mechanistically required for the altered antibiotic susceptibility phenotype or serves primarily as a marker of broader envelope stress.

Standard single-species susceptibility tests would not have predicted the highly altered antibiotic susceptibility states observed for Pa survivors in mixed-species iron-restricted biofilms, which is a key finding of this study. While polymicrobial biofilms are often more tolerant than monospecies biofilms (7, 68), the increased survival phenotype described here was not universal across antibiotic classes, as colistin remained broadly bactericidal in both single- and mixed-iron restricted macrocolonies. Although L-Ara4N addition can decrease polymyxin susceptibility (42, 60), the extent and kinetics of *arn*-dependent remodeling in this setting may be insufficient to counter rapid colistin-mediated membrane disruption at the supralethal doses used, or any partial protection may be overwhelmed. Additionally, because *arnT*, *siaD*, and *mfsC* were selected based on unique upregulation during mixed-iron restricted growth, altered colistin susceptibility in certain mutant backgrounds under single-iron restricted conditions may reflect secondary effects, such as envelope remodeling or pleiotropic stress responses, rather than disruption of the mixed-iron-restricted-specific protection mechanism itself. This class specificity suggests that some antibiotics can still circumvent the induced defenses, with potential relevance for treating polymicrobial infections where β-lactams or fluoroquinolones underperform.

**Fig 5:**
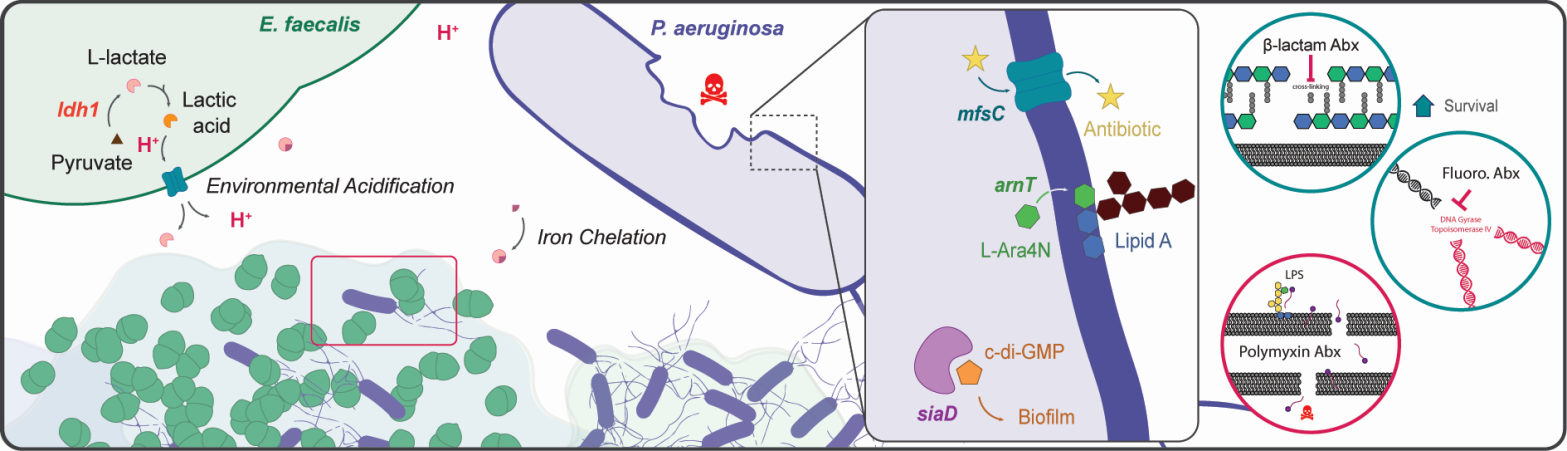
Model of *E. faecalis*-mediated antagonism and antibiotic susceptibility in *P. aeruginosa* under iron restriction. Schematic depicting the antagonistic interaction between *E. faecalis* (Ef; green) and *P. aeruginosa* (Pa; purple) in mixed-species macrocolony biofilms grown under iron-restricted conditions. Ef ferments pyruvate to lactic acid via *ldh1*, releasing protons (H^+^) and lactate that acidify the local environment and chelate iron, restricting its availability to Pa and inhibiting its growth. Within this Ef-altered, low-iron environment, surviving Pa cells exhibit a transcriptionally reprogrammed state characterized by upregulation of *arnT*, *siaD*, and *mfsC* and requiring PmrA, regulator of *arnT* (not depicted). ArnT drives L-Ara4N modification of lipid A, contributing to outer-membrane remodeling and decreased β-lactam susceptibility. SiaD increases intracellular c-di-GMP to promote decreased biofilm-associated antibiotic susceptibility, while MfsC enhances efflux. Together, these pathways reduce susceptibility to β-lactams and fluoroquinolones but not to polymyxins (colistin). This cooperative antagonism yields a small surviving Pa subpopulation with enhanced antibiotic survival under infection-relevant iron-limiting conditions.

The macrocolony biofilm model on defined medium used in this study is necessarily a simplified approximation of infection-relevant iron restriction and does not capture the full spatial, immunologic, and nutrient complexity of chronic infection niches. Nonetheless, these findings are likely relevant to settings where Pa and Ef co-colonize and where local iron limitation and fermentation-associated acidification are prominent. The pathways implicated here, *arn*-dependent lipid A modification, SiaD-mediated c-di-GMP signaling, and MfsC-linked efflux, offer potential points of therapeutic leverage. Agents that inhibit c-di-GMP signaling or diguanylate cyclases could reduce matrix-associated tolerance (68), while strategies targeting L-Ara4N addition to lipid A (PmrAB/ArnT) or otherwise perturbing envelope charge could resensitize Pa (42, 60, 65, 90). Modulating Ef lactate production or neutralizing acidification may also reduce the likelihood that Pa enters this tolerant state (33). More broadly, our findings suggest that managing polymicrobial infections may require strategies that disrupt beneficial interspecies interactions in addition to directly targeting pathogens (12, 68, 91). These findings support a model in which Ef-imposed stress under iron restriction drives a macrocolony-associated Pa survival state characterized by metabolic downshifting, altered iron-response prioritization, and a multifactorial defense involving envelope remodeling, biofilm regulatory state, and efflux that promotes survival against β-lactams and ciprofloxacin while leaving Pa vulnerable to colistin, consistent with the working model summarized in Fig. 5.

## MATERIALS AND METHODS

### Bacterial strains and growth conditions

Bacterial strains used are described in Table S5. Unless otherwise indicated, all experiments were conducted with *E. faecalis* strain OG1RF and *P. aeruginosa* strain PADP6, a 2,2′-dipyridyl-resistant strain of PAO1 used in previous work to study *P. aeruginosa* in an iron-restricted model (33). Strains were grown statically at 37°C from single colonies on tryptic soy broth (TSB; Sigma-Aldrich, USA) agar (Difco BD, USA) in 6 mL of TSB. Overnight cultures were harvested at 4,300 × *g* for 5 min, washed with sterile phosphate-buffered saline (PBS; Sigma-Aldrich, USA), resuspended in PBS, and then adjusted to 1 × 10^8^ CFU/mL for further use. When required, bacterial selection and differentiation were performed on TSB agar supplemented with either 25 µg/mL rifampicin (Sigma-Aldrich, USA) for *E. faecalis* or 100 µg/mL ampicillin (A0839 BioChemica; AppliChem GmbH, Darmstadt, Germany) for *P. aeruginosa*. For iron-restricted media, TSB agar supplemented with 10 mM glucose (Sigma-Aldrich, USA) was used with a final concentration of 3 mM 2,2′-dipyridyl (D216305; Sigma-Aldrich, USA), prepared from stock at 100 mg/mL in ethanol.

### *P. aeruginosa* strain construction

Gene deletions were constructed by two-step allelic exchange using vector pEXG2 (92). Briefly, pEXG2 vectors carrying the desired mutation were introduced into *P. aeruginosa* by conjugation from the diaminopimelic acid auxotroph *E. coli* strain WM3064. Merodiploids were selected on gentamicin, sucrose counter selection was used to select for double crossovers, and mutants carrying the desired mutation were screened by PCR. The primers used to generate deletion alleles of *mfsC*, *pmrA*, and *siaD* with upstream and downstream flanking regions of the target locus in the *P. aeruginosa* PADP6 genome by overlap extension PCR are listed in Table S6. These deletion alleles were designed to retain the start, stop, and five to seven codons to avoid potential polar effect. Plasmid inserts were verified by Sanger sequencing. The pΔ*arnT* vector used to generate the *arnT* deletion was described previously (93).

### MIC determination

MICs were determined in tryptic soy broth with glucose, described above, at 37°C by broth microdilution in accordance with Clinical and Laboratory Standards Institute guidelines using inoculum from static culture in TSB (94). All MICs were determined at least four times independently using the following concentration ranges (µg/mL): ampicillin, 20.8–1,333.3; cefepime, 0.167–10.667; ciprofloxacin, 0.042–2.667; and colistin, 0.167–10.667.

### Macrocolony biofilm assays

Macrocolony biofilm growth assays were performed by adding 1.5 mL of 3 mM 22D TSBG agar, described above, to wells of a sterile 12-well plate (BDAA353043; Corning, USA). Bacterial inocula under single-species (5 µL inoculum) or mixed-species (10 µL, 1:1 mixture) conditions were prepared as described above and spotted with one replicate per well onto the prepared agar. After drying, the plates were incubated at 37°C for 24 h before being harvested by washing twice with 1 mL of PBS. Harvested macrocolonies were then serially diluted in a 96-well plate, and 5 µL was plated on selection media as described above. After 24-h incubation at 37°C, CFUs were enumerated to determine the CFU/mL.

### Macrocolony RNA extraction

RNA-seq was performed on macrocolonies grown as described above after 24-h incubation on TSBG agar with or without 2 or 3 mM 22D in triplicate. The macrocolonies were harvested by washing twice with 0.7 mL of 1:2 PBS:bacterial RNAprotect (Qiagen, Germany) and then incubating at room temperature for 5 min. The samples were then centrifuged at 5,000 × *g* for 10 min, the supernatant was discarded, and the pellet was resuspended in 200 µL of lysis solution (1× TE, 20 mg/mL lysozyme [A37110001, Applichem], and 100 U/mL Ready-Lyse [E0057-D3, Biosearch Technologies]) before the addition of Proteinase K (15 µL; PA4850, Sigma-Aldrich). The samples were then incubated at 37°C for 30 min on a heat block before the addition of 1 mL of TRIzol reagent (Invitrogen, USA). At this point, samples could either be stored at −80°C or further processed. RNA extraction was performed by taking the samples in TRIzol reagent and adding 200 µL of chloroform per 1 mL TRIzol. Tubes were vigorously shaken for 15 s, then incubated for 3 min at room temperature. Samples were then centrifuged at 12,000 × *g* for 15 min at 4°C, and the top aqueous phase was transferred to a new 1.5 mL tube. Ethanol (80%) was added (550 µL EtOH/700 µL aqueous phase) and transferred, 700 µL at a time, to an RNeasy Mini Spin column (71404, Qiagen) for centrifugation at 8,000 × *g* for 15 s. Buffer RW1 was added (350 µL), and centrifugation was repeated. A prepared DNase mixture (10 µL DNase I with 70 µL Buffer RDD per sample, 79254, Qiagen) was added to the columns (80 µL) for on-column DNase digestion and incubated at room temperature for 15 min. Then, 350 µL of Buffer RW1 was added, followed by centrifugation. Next, 500 µL of Buffer RPE was added and centrifuged. This step was repeated with 500 µL of Buffer RPE, followed by centrifugation for 2 min at 8,000 × *g*. RNA was finally eluted into 45 µL of RNase-free water, repeating with the initial eluate. A second DNase digestion was performed with RQ1 DNase (8 U/10 µg RNA, M6101, Promega) in the presence of RNasin Plus RNase Inhibitor (8 U/10 µg RNA, N2611, Promega) for 30 min at 37°C and stopped with RQ1-DNase stop solution. After the addition of RLT buffer supplemented with β-mercaptoethanol, RNA cleanup was performed using an RNeasy Mini Spin column as described above. Purified RNA was quantified using a spectrophotometer (Ultrospec 3100 pro, Amersham Biosciences), and RNA quality was assessed using a 2100 Bioanalyzer system (Agilent).

### RNA sequencing and analysis

RNA was sequenced by the iGE3 Genomics Platform of the University of Geneva as paired-end 100 bp reads on a NovaSeq 6000 instrument (Illumina) after library preparation using the Ribo-Zero Plus rRNA Depletion kit (Illumina). The amount of libraries prepared from mixed-species samples under restriction was increased 10 times in the sequenced pool to account for the low *P. aeruginosa* CFU numbers in these samples. RNA sequencing data as fastq.gz files were processed using a custom Python pipeline, where paired-end reads were aligned to the reference genome (*E. faecalis* OG1RF, NCBI accession No. CP002621; *P. aeruginosa* PAO1, NCBI accession No. NC_002516) using BWA-MEM (version 0.7.18), the resulting BAM files were sorted using Samtools (version 1.20), and gene-level quantification was performed with HTSeq-count (version 2.0.7) using coding sequences and locus tags as identifiers, with a minimum alignment quality threshold of 10. Raw feature counts from all samples were aggregated into a single CSV file for downstream analysis. Raw feature count data were then imported into R (version 4.3.1) with RStudio (version 2023.12.1+402) and preprocessed for differential gene expression analysis using edgeR (version 4.0.16) (95). The data were filtered to remove genes with low expression levels, and normalization was performed using edgeR’s trimmed mean of M-values method to account for differences in library sizes. For each condition, data were modeled using a generalized linear model approach. Dispersion was estimated, and differential expression was assessed using a likelihood ratio test to compare expression between groups. Gene-level results were then annotated with functional information, and the top differentially expressed genes were extracted and saved for further analysis. BioCyc (96) was used for pathway analysis and to aid in figure generation. Table S8 summarizes the mapping statistics for all RNA-sequencing samples.

### Principal component analysis

For the principal component analysis (Fig. 3A), a gene-by-comparison matrix of log_2_FC values was constructed from the edgeR differential expression results described above. Genes were included if they were significantly differentially expressed (FDR ≤ 0.05) in at least one of the three pairwise comparisons (single-IR vs single-IRp, mixed-IR vs single-IR, and mixed-IRp vs single-IRp). Log_2_FC values of genes not significantly differentially expressed in a given comparison were set to 0. This resulting matrix was then subjected to PCA using the prcomp function in R with centering and scaling enabled, and each point in Fig. 2A represents the log_2_FC profile of one of the three comparisons.

### Macrocolony antibiotic susceptibility assays

Macrocolony antibiotic susceptibility testing was performed as described for macrocolony growth assays, with the addition of 24-h antibiotic exposure before harvesting. Macrocolonies grown for 24 h were treated with 1 mL of antibiotic solution in PBS, or a PBS control, added gently to the well to prevent disrupting the biofilm. After an additional 24-h incubation at 37°C with antibiotic, the solution was homogenized in the well on top of the agar, removed to a microcentrifuge tube, and the well was washed with an additional 1 mL of PBS. For dispersal measurements (as in Fig. 2), the supernatant overlay was collected for CFU determination without disrupting the macrocolony prior to harvesting the remaining macrocolony fraction. The harvested sample was centrifuged at maximum speed for 5 min. The supernatant was discarded, and the pellet was washed with 1 mL of PBS. The centrifugation was repeated, and finally, the pellet was resuspended in 1 mL of PBS for dilution plating as previously described. The final CFU/mL output of this assay is reflective of the total bacteria harvested from both biofilm-associated and potentially PBS-dispersed cells that occur during the 24-h antibiotic treatment. The antibiotic-treated condition was normalized to the PBS control to determine the percent survival following antibiotic treatment, calculated as (CFU after 24-h antibiotic exposure/CFU after 24-h PBS [average]) × 100, using the matched PBS control of the same biofilm type (single- or mixed-species) performed in parallel. A value of 100% indicates no change relative to PBS, values <100% indicate antibiotic-mediated killing, and values >100% indicate more CFU recovery after antibiotic treatment than after PBS. Antibiotics used with preparation details can be found in Table S7. For Fig. 1C and 4, percent survival values were pooled from independent experiments, and WT control data sets used for comparison with mutant strains in Fig. 4 are the same WT macrocolonies shown in Fig. 1C.

### Statistical analysis and software used

Statistical analyses were performed using GraphPad Prism (version 10.6.1; GraphPad Software, Boston, MA, USA). The specific tests applied to each data set, including unpaired two-tailed *t*-tests, one-way ANOVA with appropriate *post hoc* tests, are described in the corresponding figure legends. Statistical significance is universally represented, unless otherwise indicated, as: ns, not significant; *, *P* < 0.05; **, *P* < 0.01; ***, *P* < 0.001; ****, *P* < 0.0001; and ND, not detected (≤limit of detection) where appropriate. For samples in which no colonies were detected, the LOD value was substituted for statistical analyses and graphing to avoid zero values that preclude log transformation and distort variance estimation. This substitution was applied consistently across all experiments. Any outlier evaluation was conducted prior to statistical testing using Grubbs’ test (extreme studentized deviate test). Figures were prepared using GraphPad Prism and Adobe Illustrator (version 26.3.1). RNA processing was performed using custom Python scripts in Visual Studio Code (version 1.87.0), with differential expression analysis conducted using R (version 4.3.1) edgeR package (version 4.0.16) within RStudio (version 2023.12.1+402). Pathway analysis shown in Fig. 3 was performed using BioCyc Pathway Analysis (96). Table S9 contains all raw CFU/mL values for generating Fig. 1B through E, 3B through E, and Fig. S1 parts 1 and 2, as well as *z*-scores for Fig. 4A.

## Data Availability

RNA sequencing data have been deposited in the National Center for Biotechnology Information (NCBI) Sequence Read Archive (SRA) database under BioProject PRJNA1365690.

## SUPPLEMENTAL MATERIAL

Figure S1 Part 1 jb.00548-25-s0001.tif Absolute CFU/mL values for macrocolony antibiotic susceptibility assays (PBS, ampicillin, cefepime).

Figure S1 Part 2 jb.00548-25-s0002.tif Absolute CFU/mL values for macrocolony antibiotic susceptibility assays (ciprofloxacin, colistin).

Supplemental material jb.00548-25-s0003.docx Tables S1 and S5-S8, descriptions for Tables S2-S4 and S9, and Fig. S1 and S2 legends.

Table S2 jb.00548-25-s0004.xlsx Differentially expressed genes (DEGs) from RNA-seq datasets.

Table S3 jb.00548-25-s0005.xlsx Venn/PCA gene sets corresponding to Fig. 3A.

Table S4 jb.00548-25-s0006.xlsx Fig. 3C pathways.

Table S9 jb.00548-25-s0007.xlsx Raw CFU/mL macrocolony values used for generating Fig. 1B-E, Fig. 2B-D, Fig. 4B-E, and Fig. S1 Part 1 and Part 2, as well as z-scores for Fig. 4A.

ASM does not own the copyrights to Supplemental Material that may be linked to, or accessed through, an article. The authors have granted ASM a non-exclusive, world-wide license to publish the Supplemental Material files. Please contact the corresponding author directly for reuse.
