# Supplementary figures and images for: Enterococcus faecalis alters antibiotic susceptibility in Pseudomonas aeruginosa mixed-species biofilms
Source: J Bacteriol. 2026 Apr 30;208(5):e00548-25. doi: 10.1128/jb.00548-25 (PMC13192264; doi:10.1128/jb.00548-25)

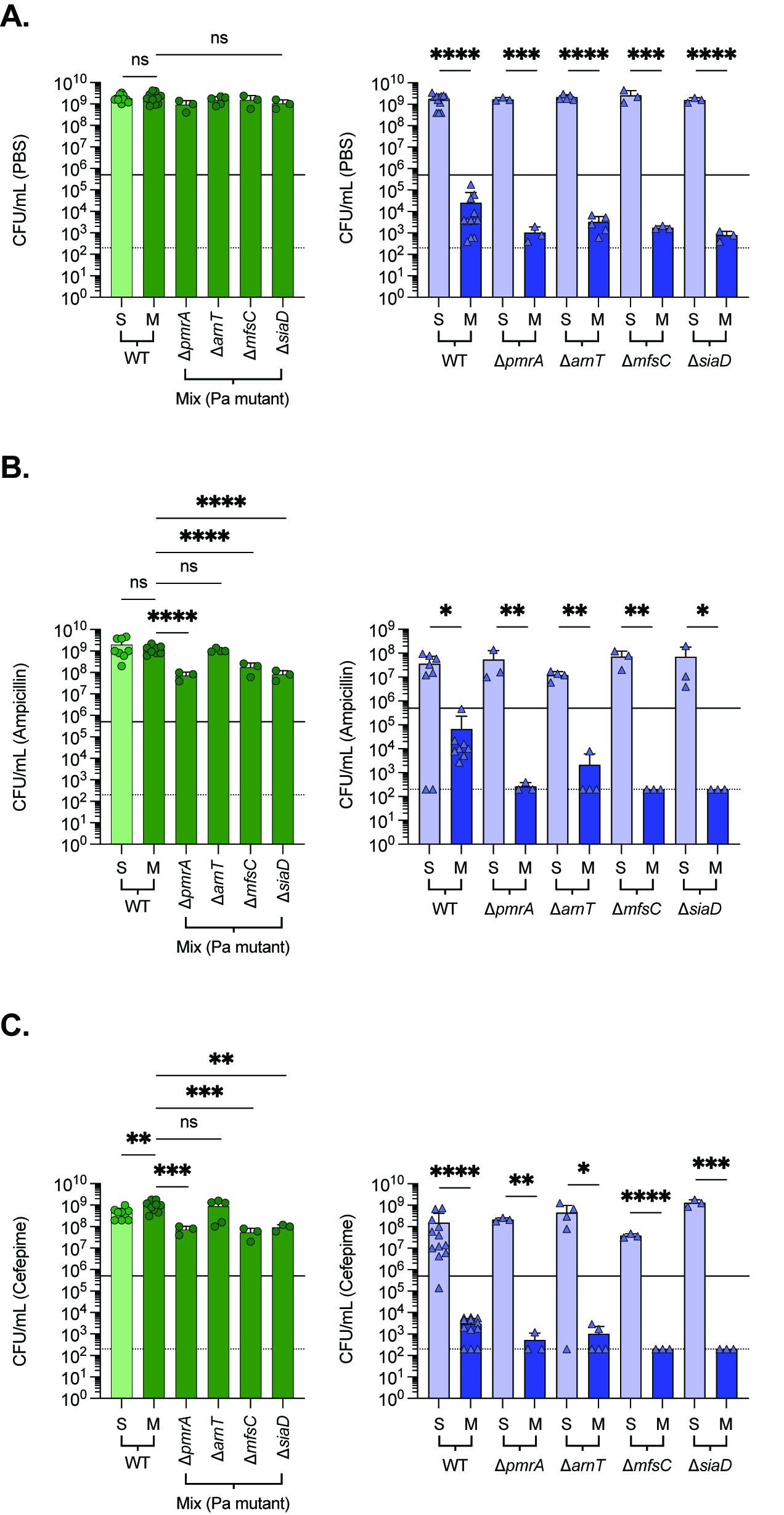

Supplement: Figure S1 Part 1 — Absolute CFU/mL values for macrocolony antibiotic susceptibility assays (PBS, ampicillin, cefepime). [file jb.00548-25-s0001.tif]

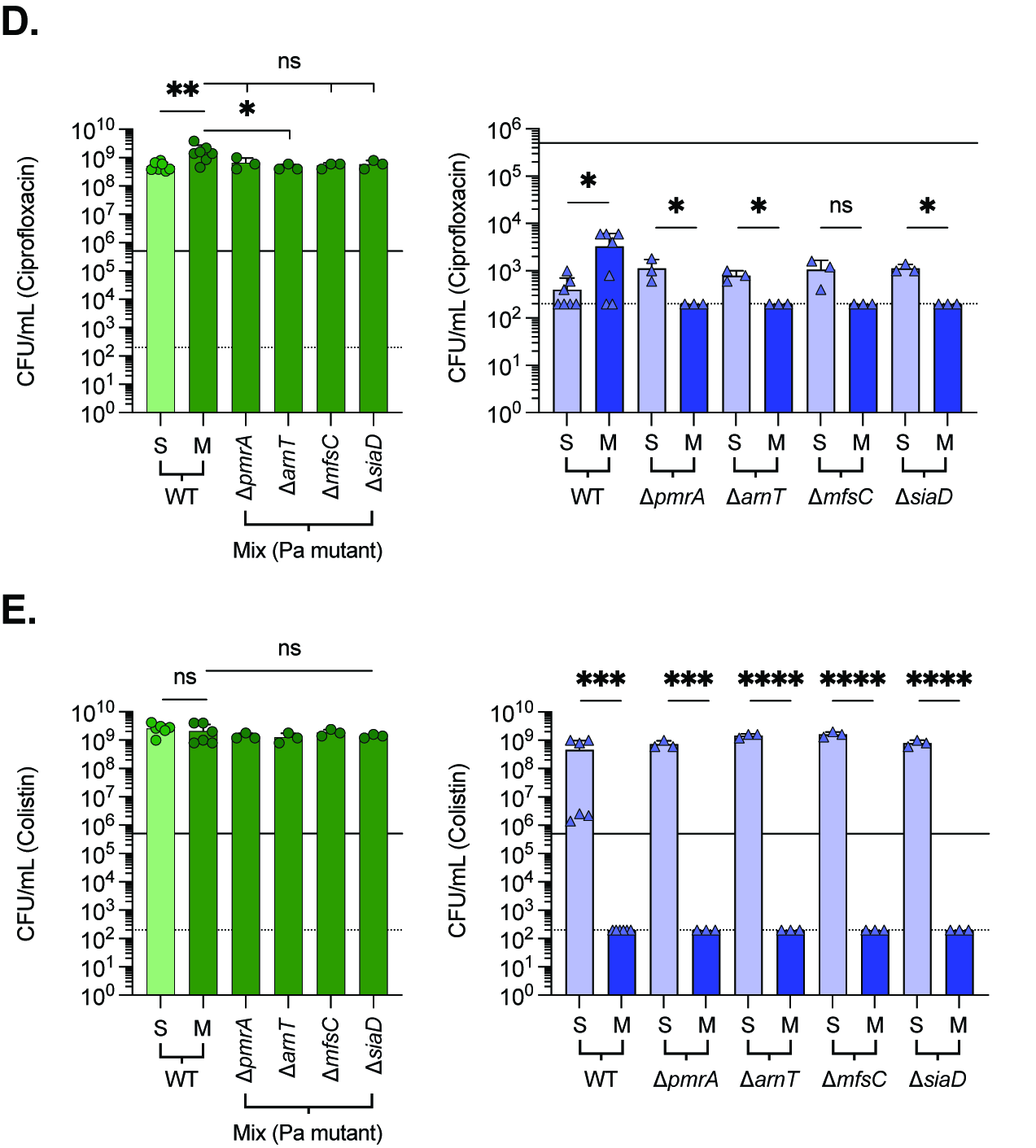

Supplement: Figure S1 Part 2 — Absolute CFU/mL values for macrocolony antibiotic susceptibility assays (ciprofloxacin, colistin). [file jb.00548-25-s0002.tif]
