# Supplementary material for: Enterococcus faecalis alters antibiotic susceptibility in Pseudomonas aeruginosa mixed-species biofilms
Source: J Bacteriol. 2026 Apr 30;208(5):e00548-25. doi: 10.1128/jb.00548-25 (PMC13192264; doi:10.1128/jb.00548-25)
Supplement: Supplemental material — Tables S1 and S5-S8, descriptions for Tables S2-S4 and S9, and Fig. S1 and S2 legends. [file jb.00548-25-s0003.docx]

**SUPPLEMENTARY MATERIALS**

**Table S1.** Planktonic MIC values for *P. aeruginosa* strains PAO1 and PADP6.

|  |  | PADP6 | | | | |
| --- | --- | --- | --- | --- | --- | --- |
| MIC* (µg/mL) | PAO1 WT | WT | *ΔpmrA* | *ΔarnT* | *ΔmfsC* | *ΔsiaD* |
| AMP | 666.7 | 1333.3 | 1333.3 | 1333.3 | 1333.3 | 1333.3 |
| CEF | 2.667 | 5.333 | 5.333 | 5.333 | 5.333 | 5.333 |
| CIP | 0.167-0.333 | 0.667 | 0.667 | 0.667 | 0.667 | 0.667 |
| COL | 0.667-1.333 | 1.333 | 1.333 | 1.333 | 1.333 | 1.333 |

*AMP = ampicillin; CEF = cefepime; CIP = ciprofloxacin ; COL = colistin

**Table S2.** Differentially expressed genes (DEGs) from RNA-seq datasets.

(SM_Table_S5_DEG.xlsx)

**Table S3.** Venn/PCA gene sets corresponding to Figure 3A. (SM_Table_S6_PCA_Venn.xlsx)

**Table S4.** Figure 3C Pathways. (SM_Table_S7_Fig3C_Pathways.xlsx)

**Table S5.** Strains used.

| Strain | Reference |
| --- | --- |
| *E. faecalis* OG1RF | (1) |
| *P. aeruginosa* PADP6 | (2) |
| *P. aeruginosa* PAO1 WT | (3) |
| PADP6 Δ*arnT* | This study |
| PADP6 Δ*mfsC* | This study |
| PADP6 Δ*pmrA* | This study |
| PADP6 Δ*siaD* | This study |

**REFERENCES**

1. Dunny GM, Brown BL, Clewell DB. 1978. Induced cell aggregation and mating in *Streptococcus faecalis*: evidence for a bacterial sex pheromone. Proceedings of the National Academy of Sciences 75:3479-3483.
2. Tan CAZ, Lam LN, Biukovic G, Soh EY, Toh XW, Lemos JA, Kline KA. Enterococcus faecalis Antagonizes Pseudomonas aeruginosa Growth in Mixed-Species Interactions. J Bacteriol. 2022 Jul 19;204(7):e0061521. doi: 10.1128/jb.00615-21. Epub 2022 Jun 27. PMID: 35758750; PMCID: PMC9295543.
3. Hentzer M, Riedel K, Rasmussen TB, Heydorn A, Andersen JB, Parsek MR, Rice SA, Eberl L, Molin S, Høiby N. 2002. Inhibition of quorum sensing in Pseudomonas aeruginosa biofilm bacteria by a halogenated furanone compound. Microbiology 148:87-102.

**Table S6.** Primers used to construct *P. aeruginosa* deletion alleles.

| **Primer** | **Sequence*** | **Purpose** |
| --- | --- | --- |
| Ext_Dn_XbaI_  del-mfsC | agtgTCTAGAgcggtccacatgggattcc | amplify mfsC deletion allele  with flanking region and clone  into pEXG2 |
| Ext_Up_HindIII_  del-mfsC | actgAAGCTTgacgacgcccgctcga | amplify mfsC deletion allele  with flanking region and clone  into pEXG2 |
| SOE_Dn_  del-mfsC | cagcatgagcgaagcctccgggcactgaggac | generate mfsC deletion allele  by overlap extension PCR |
| SOE_Up_  del-mfsC | cagtgcccggaggcttcgctcatgctggcagg | generate mfsC deletion allele  by overlap extension PCR |
| Ext_Dn_XbaI_  del-pmrA | agtgTCTAGAtagaacagcagcaggttcatgg | amplify pmrA deletion allele  with flanking region and clone  into pEXG2 |
| Ext_Up_HindIII_  del-pmrA | agtgAAGCTTtggcacgaaggtcacctg | amplify pmrA deletion allele  with flanking region and clone  into pEXG2 |
| SOE_Dn_  del-pmrA | catgagaatactgctggccccctgaaaactgcctaccg | generate pmrA deletion allele  by overlap extension PCR |
| SOE_Up_  del-pmrA | cggtaggcagttttcagggggccagcagtattctcatggc | generate pmrA deletion allele  by overlap extension PCR |
| Ext_Dn_XbaI_  del-siaD | agtgTCTAGAgcgcagtaccgcttcagc | amplify siaD deletion allele with flanking region and clone into pEXG2 |
| Ext_Up_HindIII_  del-siaD | agtcAAGCTTcatcatgagtgacctgcacataccc | amplify siaD deletion allele with flanking region and clone into pEXG2 |
| SOE_Dn_  del-siaD_new | gagcgcatcgcccgctgagcctcctcagg | generate siaD deletion allele  by overlap extension PCR |
| SOE_Up_  del-siaD_new | ggaggctcagcgggcgatgcgctc | generate siaD deletion allele  by overlap extension PCR |

*N.B. Restriction sites are in caps.

**Table S7.** Antibiotics used for macrocolony antibiotic susceptibility testing.

| **Antibiotic** | **Class/Mechanism of Action** | **Vendor** | **Stock Solution** |
| --- | --- | --- | --- |
| Ampicillin | ß-lactam, peptidoglycan biosynthesis | Biochemica; A0839,0025 | 100 mg/mL (dH2O) |
| Cefepime | ß-lactam, peptidoglycan biosynthesis | Thermo Fisher Scientific; J66237 | 100 mg/mL (DMSO) |
| Ciprofloxacin | Fluoroquinolone, DNA replication | Sigma-Aldrich; 17850-5G-F | 10 mg/mL (dH2O) |
| Colistin | Polymyxin, cell membrane disruption | Thermo Fisher Scientific; J60915 | 50 mg/mL (dH2O) |

**Table S8**. Summary of sequencing depth and read alignment to the *P. aeruginosa* PAO1 and *E. faecalis* OG1RF genomes across all RNA-sequencing samples.

|  | **Total Reads** | **Mapped Reads** | |
| --- | --- | --- | --- |
| **Sample** |  | **Pa** | **Ef** |
| E0A | 1.47 x 10^7^ | - | 1.47 x 10^7^ |
| E0B | 1.69 x 10^7^ | - | 1.69 x 10^7^ |
| E0C | 1.66 x 10^7^ | - | 1.66 x 10^7^ |
| E3A | 1.81 x 10^7^ | - | 1.81 x 10^7^ |
| E3B | 2.00 x 10^7^ | - | 1.99 x 10^7^ |
| E3C | 1.84 x 10^7^ | - | 1.84 x 10^7^ |
| P0A | 1.67 x 10^7^ | 1.66 x 10^7^ | - |
| P0B | 1.69 x 10^7^ | 1.68 x 10^7^ | - |
| P0C | 2.30 x 10^7^ | 2.30 x 10^7^ | - |
| P3A | 1.63 x 10^7^ | 1.62 x 10^7^ | - |
| P3B | 1.87 x 10^7^ | 1.86 x 10^7^ | - |
| P3C | 2.29 x 10^7^ | 2.28 x 10^7^ | - |
| EP0A | 1.80 x 10^7^ | 1.26 x 10^7^ | 5.58 x 10^6^ |
| EP0B | 1.93 x 10^7^ | 1.41 x 10^7^ | 5.29 x 10^6^ |
| EP0C | 1.83 x 10^7^ | 1.31 x 10^7^ | 5.33 x 10^6^ |
| EP3A | 1.59 x 10^8^ | 1.18 x 10^6^ | 1.58 x 10^8^ |
| EP3B | 1.52 x 10^8^ | 1.39 x 10^6^ | 1.51 x 10^8^ |
| EP3C | 1.33 x 10^8^ | 8.04 x 10^5^ | 1.33 x 10^8^ |

**Table S9.** Tables containing raw CFU/mL macrocolony values used for generating **Figure 1B-E**, **Figure 4B-E**, **Figure S1 Part 1** and **Part 2**, as well as z-scores for **Figure 4A**. (SM_Table_S9_CFU.xlsx)

**Figure S1. Part 1. Absolute CFU/mL values for macrocolony antibiotic susceptibility assays (PBS, ampicillin, cefepime).** Bar graphs show absolute CFU/mL recovered from 24-hour (h) macrocolonies grown under iron-restricted conditions (3 mM 2,2′-dipyridyl in tryptic soy broth (TSB) agar + 10 mM glucose; 22D TSBG) and then exposed for 24-h to PBS (**A**), ampicillin (50 mg/mL) (**B**), or cefepime (1 mg/mL) (**C**). Data are shown separately for *E. faecalis* (Ef; green) and *P. aeruginosa* (Pa; purple) grown as single- or mixed-species (1:1) macrocolonies. The solid horizontal line marks the inoculum (5 × 10^5^ CFU/mL) and the dotted line indicates the limit of detection (LOD, 200 CFU/mL). Bars denote mean ± standard deviation (SD) with individual biological replicates (*n*) overlaid. Statistical significance determined after log_10_ transformation of CFU/mL values via unpaired two‑tailed *t* tests or one-way ANOVA for Ef + Pa mutants; ns, not significant; *, *P*<0.05; **, *P*<0.01; ***, *P*<0.001; ****, *P*<0.0001. Data shown correspond to the CFU/mL values used to produce the percent survival plotted in **Figure 4** and **Figure 1C**.

**Figure S1. Part 2. Absolute CFU/mL values for macrocolony antibiotic susceptibility assays (ciprofloxacin, colistin).** As in **Figure S1,** **Part 1**, absolute CFU/mL after 24-h antibiotic exposure under iron-restricted macrocolony growth are shown for ciprofloxacin (1 mg/mL) (**D**) and colistin (10 µg/mL) (**E**). Ef (green) and Pa (purple) are plotted for single vs mixed (1:1) biofilms. Inoculum (solid) and LOD (dotted) lines are shown as in **Figure S1,** **Part 1**. Bars denote mean ± standard deviation (SD) with individual biological replicates (*n*) overlaid. Statistical significance determined after log_10_ transformation of CFU/mL values via unpaired two‑tailed *t* tests or one-way ANOVA for Ef + Pa mutants; ns, not significant; *, *P*<0.05; **, *P*<0.01; ***, *P*<0.001; ****, *P*<0.0001. Data shown correspond to the CFU/mL values used to produce the percent survival plotted in **Figure 4** and **Figure 1C**.
